# Supplementary material for: Cerebral blood flow and cerebrovascular reactivity are preserved in a mouse model of cerebral microvascular amyloidosis
Source: eLife. 2021 Feb 12;10:e61279. doi: 10.7554/eLife.61279 (PMC7880694; doi:10.7554/eLife.61279)
Supplement: Transparent reporting form [file elife-61279-transrepform.docx]

***eLife’s* transparent reporting form**

We encourage authors to provide detailed information *within their submission* to facilitate the interpretation and replication of experiments. Authors can upload supporting documentation to indicate the use of appropriate reporting guidelines for health-related research (see [EQUATOR Network](about:blank)), life science research (see the BioSharing Information Resource), or the [ARRIVE guidelines](about:blank) for reporting work involving animal research. Where applicable, authors should refer to any relevant reporting standards documents in this form.

If you have any questions, please consult our Journal Policies and/or contact us: [editorial@elifesciences.org](about:blank).

**Sample-size estimation**

- You should state whether an appropriate sample size was computed when the study was being designed
- You should state the statistical method of sample size computation and any required assumptions
- If no explicit power analysis was used, you should describe how you decided what sample (replicate) size (number) to use

Please outline where this information can be found within the submission (e.g., sections or figure legends), or explain why this information doesn’t apply to your submission:

To estimate the sample size for cohort 1 in our study, we started with the results of Park *et al.,* (Stroke 2014). In that study, the authors showed with a sample size of 5 animals, that at 3 months of age, Transgenic Swedish Dutch Iowa (Tg‑SwDI) mice have an approximately 35 % mean CBF increase upon hypercapnia, versus 65 % in the wild type (WT) controls. The standard deviation was approximately 12 % in both groups (these numbers are estimated from the bar graphs in figure 1 of Park *et al.*).

We did not want to assume normality of the to be acquired data, given that normality testing is problematic with small sample sizes. We therefore computed our sample size for a two-sided Mann-Whitney U test, with an alpha of 0.05 and power of at least 80 %.

Given the effect size of 30 percentage points difference between Tg‑SwDI and wild type mice, and a standard deviation of 12 % in both groups, 4 animals would be needed per group. We used G*Power software for the sample size calculation (Faul *et al.,* Behavior Research Methods 2007).

However, if we would measure slightly higher standard deviations, this could easily increase the required sample size. Furthermore, it could be possible that drop-outs would occur. As such, we did not want to risk working with 4 animals per group only. Furthermore, we did not know how the differences in imaging modalities (ASL-MRI instead of LDF) and anesthesia (isoflurane versus urethane and alpha-chloralose) would affect the effect size and/or standard deviation. Therefore, we chose to increase our sample size to 9.

We considered this information too detailed for the manuscript, and as such this information is not outlined in the manuscript.

Cohort 2 was a proof-of-principle experiment, to validate whether large differences are present when using different imaging modalities (ASL-MRI vs LDF). This might then explain the discrepancy in outcome between our study and the study of Park *et al*. Thus, no specific power test was performed for this cohort.

**Replicates**

- You should report how often each experiment was performed
- You should include a definition of biological versus technical replication
- The data obtained should be provided and sufficient information should be provided to indicate the number of independent biological and/or technical replicates
- If you encountered any outliers, you should describe how these were handled
- Criteria for exclusion/inclusion of data should be clearly stated
- High-throughput sequence data should be uploaded before submission, with a private link for reviewers provided (these are available from both GEO and ArrayExpress)

Please outline where this information can be found within the submission (e.g., sections or figure legends), or explain why this information doesn’t apply to your submission:

This study was only carried out once, and none of the data has been excluded. The different animals in each cohort are defined as biological replication. There are no technical replications in this study. Given that the repeated measurements are acquired at increasing age of the animals, these are also not considered as technical replications.

**Statistical reporting**

- Statistical analysis methods should be described and justified
- Raw data should be presented in figures whenever informative to do so (typically when N per group is less than 10)
- For each experiment, you should identify the statistical tests used, exact values of N, definitions of center, methods of multiple test correction, and dispersion and precision measures (e.g., mean, median, SD, SEM, confidence intervals; and, for the major substantive results, a measure of effect size (e.g., Pearson's r, Cohen's d)
- Report exact p-values wherever possible alongside the summary statistics and 95% confidence intervals. These should be reported for all key questions and not only when the p-value is less than 0.05.

Please outline where this information can be found within the submission (e.g., sections or figure legends), or explain why this information doesn’t apply to your submission:

The statistical analysis methods and multiple-testing correction are described in the “Statistical testing” paragraph in the materials and methods section. The animal numbers are described in the “Animals” paragraph in the materials and methods section. Raw data is outlined in figures 3, 4 and 5, as well as in figure 3-supplement 1. The results section elaborately describes the medians, confidence intervals, exact p-values and summary statistics for the major substantive results.

(For large datasets, or papers with a very large number of statistical tests, you may upload a single table file with tests, Ns, etc., with reference to sections in the manuscript.)

**Group allocation**

- Indicate how samples were allocated into experimental groups (in the case of clinical studies, please specify allocation to treatment method); if randomization was used, please also state if restricted randomization was applied
- Indicate if masking was used during group allocation, data collection and/or data analysis

Please outline where this information can be found within the submission (e.g., sections or figure legends), or explain why this information doesn’t apply to your submission:

Randomization was not applicable, as no treatment was applied.

Masking was not used during this study. However, given that the ASL-MRI and LDF measurements of CBF are likely not dependent on any user bias, it is expected that the lack of masking will not have biased the outcome.

**Additional data files (“source data”)**

- We encourage you to upload relevant additional data files, such as numerical data that are represented as a graph in a figure, or as a summary table
- Where provided, these should be in the most useful format, and they can be uploaded as “Source data” files linked to a main figure or table
- Include model definition files including the full list of parameters used
- Include code used for data analysis (e.g., R, MatLab)
- Avoid stating that data files are “available upon request”

Please indicate the figures or tables for which source data files have been provided:

Raw ASL-MRI data for figures 2 till 5 are uploaded to the external open source server, as described in the manuscript.
